# Insights into Human Astrocyte Response to H5N1 Infection by Microarray Analysis

**DOI:** 10.3390/v7052618

**Published:** 2015-05-22

**Authors:** Xian Lin, Ruifang Wang, Jun Zhang, Xin Sun, Zhong Zou, Shengyu Wang, Meilin Jin

**Affiliations:** 1 State Key Laboratory of Agricultural Microbiology, Huazhong Agricultural University, Wuhan 430070, China; E-Mails: yoya12@163.com (X.L.); wrf138493@126.com (R.W.); zhangjun851029@126.com (J.Z.); coolxin@foxmail.com (X.S.); zz19841024@126.com (Z.Z); sywang2012@126.com (S.W.); 2 Laboratory of Animal Virology, College of Veterinary Medicine, Huazhong Agricultural University, Wuhan 430070, China; 3 Key Laboratory of Development of Veterinary Diagnostic Products, Ministry of Agriculture, College of Veterinary Medicine, Huazhong Agricultural University, Wuhan 430070, China

**Keywords:** H5N1, astrocytes (U251), microarray analysis, innate immune, pro-inflammatory response, CNS disorder

## Abstract

Influenza virus infects not only the respiratory system but also the central nervous system (CNS), leading to influenza-associated encephalopathy and encephalitis. Astrocytes are essential for brain homeostasis and neuronal function. These cells can also be infected by influenza virus. However, genome-wide changes in response to influenza viral infection in astrocytes have not been defined. In this study, we performed gene profiling of human astrocytes in response to H5N1. Innate immune and pro-inflammatory responses were strongly activated at 24 h post-infection (hpi). Antiviral genes, as well as several cytokines and chemokines, including CXCL9, CXCL10, and CXCL11, were robustly induced. Phosphorylation of p65 and p38 can be activated by viral infection, suggesting their potential critical roles in H5N1-induced pro-inflammatory response. Moreover, H5N1 infection significantly upregulated the gene expressions related to the neuroactive ligand-receptor interaction pathway at 24 hpi, such as MC2R, CHRNG, P2RY13, GABRA1, and HRH2, which participant in synaptic transmission and may take part in CNS disorders induced by H5N1 infection. Targeting key components of innate immune response and the neuroactive ligand-receptor interaction pathway may provide a strategy to control H5N1-induced encephalopathy and encephalitis. This research can contribute to the understanding of H5N1 pathogenesis in astrocytes.

## 1. Introduction

Currently, one of the greatest worldwide pandemic threats is posed by the highly pathogenic family of avian H5N1 influenza A viruses. Although most infections primarily affect the respiratory system, a substantial amount of evidence demonstrates that influenza viral insults can directly happen in brains [1,2,3,4,5,6]. Recently, avian and human H5N1 infections have exhibited acute neurologic signs, ranging from mild encephalitis to motor disturbances and even comas [7,8]. H5N1 infections can also initiate central nervous system (CNS) disorders and induce a long-lasting inflammatory responses in the brain [9,10]. Although the development of influenza-associated encephalopathy and encephalitis is not completely elucidated, direct viral damage and immunopathologic injury, which are mediated by the activated innate CNS immune system, are suggested as two important contributors [11].

Astrocytes, the most abundant cells in the CNS, can produce cytokines and neurotrophic factors and are essential for brain homeostasis and neuronal function [12,13]. These cells are also an integral part of the blood-brain barrier (BBB). Previous studies suggested that influenza viruses can infect astrocytes and induce the pro-inflammatory cytokine response, as well as apoptosis [11,14]. Recent studies have demonstrated that aberrant host innate immune responses to H5N1 may be responsible for pathogenicity [15,16]. Nevertheless, very little mechanistic data are available regarding host response to H5N1 infection in astrocytes. In the present study, a functional genomics approach was used to investigate the comprehensive host response to H5N1 infection in U251, a human astrocyte cell line. Microarray data showed that H5N1 infection can significantly modulate several pathways, including innate immune responses (toll-like receptor, RIG-I-like receptor, and interferon (IFN) signaling pathways), pro-inflammatory responses (chemokine receptors bind chemokines and cytokine-cytokine receptor interaction) and neuroactive ligand-receptor interaction, especially at 24 h post-infection (hpi), compared with control infection. Further study demonstrated that both NF-κB and p38 MAPK can be activated by H5N1, and inhibit p38 kinase by SB203580 significantly reduced virus-induced cytokines produce, suggesting that the p38 MAPK pathway may play important role in pro-inflammatory response to H5N1 infection. The results suggested that over-activated IFN signaling and intense IFN-stimulated genes (ISGs) produce and excessive proinflammatory response contributed to the immunologic injury of U251 by H5N1 infection. Furthermore, the modulated genes related to neuroactive ligand-receptor interaction in astrocytes could be related to the neural signaling disorder and internal Ca^2+^ elevation in CNS caused by H5N1. To our knowledge, this research is the first microarray analysis performed in H5N1-infected astrocytes and can provide a basis for understanding the H5N1 pathogenicity in astrocytes.

## 2. Materials and Methods

### 2.1. Cells and Virus

Human astrocyte cells U251 and Madin-Darby canine kidney (MDCK) cells were obtained from China Center for Type Culture Collection (CTCC, Wuhan, China),maintained in Dulbecco’s modified Eagle’s medium (DMEM), supplemented with 10% heat-inactivated fetal bovine serum (FBS) (HyClone, Logan, UT, USA), and incubated in 37 °C humidified incubator with 5% CO_2_.A/duck/Hubei/hangmei01/2006(H5N1) (HM/06) isolated from duck brain tissues[17], which can cause neurovirulence and mortality in ducks, was propagated in nine-day-old specific pathogen-free embryonated eggs and titrated in MDCK cells by common plaque assays. To determine the viral growth curve, U251 cells were inoculated with HM/06 at multiplicity of infection (MOI) of 1.0 in six-well plates. After 40 min adsorption, cells were washed once with warm phosphate-buffered saline (PBS) and then incubated in DMEM containing 0.2% FBS at 37 °C. At time points of 6, 12, and 24 hpi, supernatants were collected and titrated by TCID_50_ in MDCK cells. All the infection experiments were performed in a Biosafety Level 3 laboratory.

### 2.2. Apoptosis Detection

U251 cells were infected with HM/06 at MOI1.0. At time points of 6, 12, and 24hpi, cells were washed with PBS, detached from culture plates with 0.25% trypsin (EDTA-free), and spun down. Cells were washed twice with cold PBS. FITC-annexin V Apoptosis Detection Kit (Biolegand, San Diego, CA, USA) was used in accordance with the manufacturer’s instructions to detect cell apoptosis via the BD FACSCalibur system.

### 2.3. Immunofluorescence Assay

U251 cells were inoculated with HM/06 at MOI1.0 in 24-well plates. The cells were then washed with warm PBS at the indicated time points post-infection and fixed with 4% formaldehyde in PBS at room temperature. After washing, cells were permeabilized in 0.1%to 0.2% Triton X-100 for 10 min and then washed again. Permeabilized cells were blocked with 2% BSA for 1 h and stained with antibodies directed against nucleoprotein (NP) of influenza virus for 1 h at 37 °C. After re-washing, cells were incubated with relevant secondary antibody for 1 h at 37 °C. After a final wash with PBS, cells were incubated with DAPI (Beyotime, Shanghai, China) for nuclear staining. Fluorescence was visualized under an Olympus IX70 microscope.

### 2.4. Agilent Microarray Experiments

U251 cells were inoculated with HM/06 at MOI1.0 or infected control. At time points of 6, 12, and 24hpi (with three replicates per time point), total RNA from virus-infected and non-infected U251 cells was extracted using TRIzol, following standard instructions (Invitrogen, Carlsbad, CA, USA). RNA integrity and concentration were evaluated by an Agilent 2100 Bioanalyzer (Agilent Technologies, Palo Alto, CA, USA). RNA labeling and hybridization were conducted using a commercial Agilent array service (Shanghaibio, Shanghai, China), following the standard one-cycle protocol in accordance with the manufacturer’s instructions. Transcriptional profiles were assessed by 4x44K Agilent whole human genome oligo microarray (with three chips per sample).Hybridization and scanning of arrays were performed in accordance with standard protocols using a G2565BA Scanner (Agilent).Raw data were extracted with Feature Extraction software 10.7 (Agilent) and normalized using the quantile algorithm in GeneSpring GX 11.0 (Agilent). ANOVA, using Benjamini-Hochberg multiple testing correction, was performed to identify the genes significantly differentially expressed (DE) (*p* < 0.05) in response to viral infection. Significantly DE genes with fold change ≥2.0 were then collected for further Gene Ontology (GO) and pathway analysis. GO and pathway over-representation analysis were conducted using the InnateDB platform [18]. Over-representation analyses were performed using default parameters (hypergeometric algorithm and Benjamini-Hochberg multiple testing correction). Results with *p* < 0.05 after multiple testing corrections were considered statistically significant. Ingenuity Pathway Analysis 5.0 (IPA) (Ingenuity Systems, Redwood City, CA, USA) was used to analyze the pathway and the Diseases and Functions Heat Map. The raw and processed data discussed in this study have been deposited in NCBI’s Gene Expression Omnibus (GEO) and are accessible through GEO series accession number GSE66597.

### 2.5. Western Blot

Briefly, after infection, cells were thoroughly washed and lysed in cell Tris lysis buffer (Cell Signaling) on ice for 45 min. The lysates were briefly sonicated and cleared by centrifugation at 12,000 rpm for 10 min at 4 °C. The lysates were further denatured by incubation for 5 min at 95 °C in loading buffer. The samples were then subjected to SDS-PAGE and transferred to nitrocellulose membranes (Whatman, Kent, UK). After blocking in 2% BSA, the membrane was reacted with primary antibodies for 2 h at room temperature, followed by HRP-conjugated secondary antibodies (Jackson ImmunoResearch Laboratories) for 1 h at room temperature. The signals were detected using Immobilon Western Chemiluminescent HRP Substrate kit (Thermo Fisher, , Waltham, MA, USA) and ChemBis (Eastwin, Beijing, China).Rabbit polyclonal anti-p38, p-p38, ERK1/2, p-ERK1/2, p65, p-p65, and RIG-I were purchased from Cell Signaling (Beverly, MA, USA), whereas mouse monoclonal anti-GAPDH was purchased from California Bioscience (Coachella, CA, USA).

### 2.6. Real-Time Quantitative RT-PCR (qRT-PCR) Assays

Total RNA of virus-infected and non-infected U251 cells was isolated using TRIzol, as described above. For some experiments, cells were pretreated with NF-κB inhibitor PDTC or p38 kinase inhibitor SB203580 (Sigma, Saint Louis, MO, USA) or mock treated before H5N1 virus challenge for 30 min. One microgram RNA was reversely transcribed in 20 μL reaction mixture containing 2μL avian myeloblastosis virus (AMV) buffer, 50 pm Olig18T, 0.5 mM dNTPs, 10 U RNase inhibitor, and 20 U AMV reverse transcriptase (TaKaRa, Otsu, Japan). Transcript expression was monitored by using SYBR Green-based RT-PCR with ABI ViiA 7 PCR system (Applied Biosystems, Foster City, CA, USA), along with corresponding primers.The expression of each specific gene was normalized to the levels of GAPDH. Changes in gene expression were calculated by *t*-test, and *p* < 0.05 was considered significant. All primers used in this study are listed in Supplementary Table S1.

### 2.7. Detection of Ca^2+^Accumulation

The culture medium of infected or control cells was changed to HBSS that contained 5 μM Fluo4-AM (Invitrogen, Carlsbad, CA, USA), an indicator of Ca^2+^ in the cytosol. After incubation at 37 °C for 30 min, cells were washed twice with HBSS. Fluorescence was analyzed via the BD FACSCalibur system.

### 2.8. Statistical Analysis

Data were expressed as means ± SEM. Significance was determined with Student *t*-test (*p* < 0.05).

## 3. Results

### 3.1. Infection of U251 Cells by HM/06

**Figure 1:**
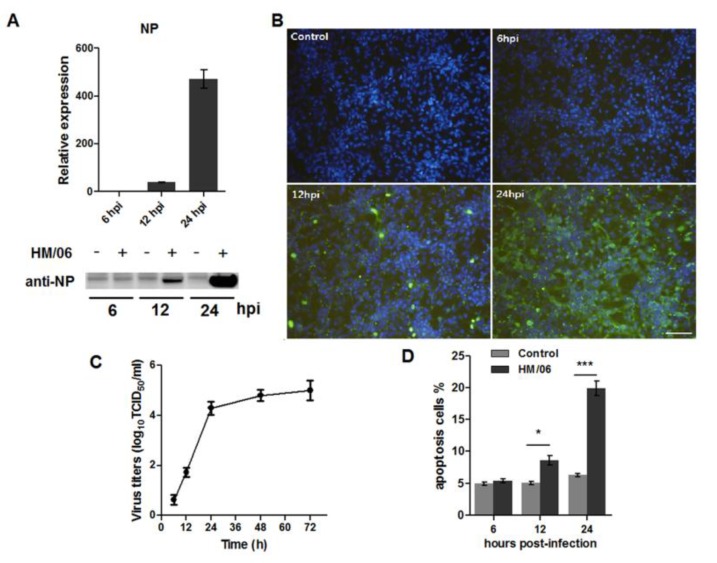
HM/06 replicated efficiently in U251 cells and induced apoptosis in U251; (**A**) U251 cells were infected by HM/06 at MOI 1.0, and cells were lysed for NP detecting by western blot at indicated times post-infection; (**B**) HM/06-infected U251 cells were fixed, permeabilized, and stained with corresponding antibodies for immunofluorescence observation with an OLYMPUSIX70microscope. Bar, 50 µm; (**C**) U251 cells were infected by HM/06 at MOI 1.0, and at indicated times post-infection, supernatants were collected and viruses titer were determined by TCID_50_ in MDCK cells. Data was showed as means ± SEM from three experiments; (**D**) U251 cells were infected by HM/06, and at 6, 12, and 24hpi, cells were washed with PBS, detached from the culture plates. Apoptosis was detected using FITC-Annexin V Apoptosis Detection Kit. Data were presentedas mean ± SEM from three experiments. Statistical significance was analyzed by student’s *t*-test. * *p* < 0.05, *** *p* < 0.001.

To evaluate infection of HM/06 in U251, we inoculated HM/06 at MOI 1.0 in U251 cells. The NP gene was monitored by RT-PCR and Western blot (Figure 1A). In addition, immunofluorescence assay was performed to better understand the infection process (Figure 1B). To further examine the viral replication kinetics at such dose of infection, supernatants from infected cells were collected at indicated times post-infection, and viruses were determined by TCID_50_ assay (Figure 1C). Together, these data demonstrated that HM/06 could efficiently replicate in U251 cells. Apoptosis induced by virus serves as part of mechanisms contributing to cellular dysfunction, withH5N1 inducing significant apoptosis in numerous cell types. In this study, apoptosis was also investigated (Figure 1D), which suggested that HM/06 could induce apparent cell apoptosis in U251 cells.

### 3.2. Gene Expression Alterations in Infected U251 Cells

**Figure 2:**
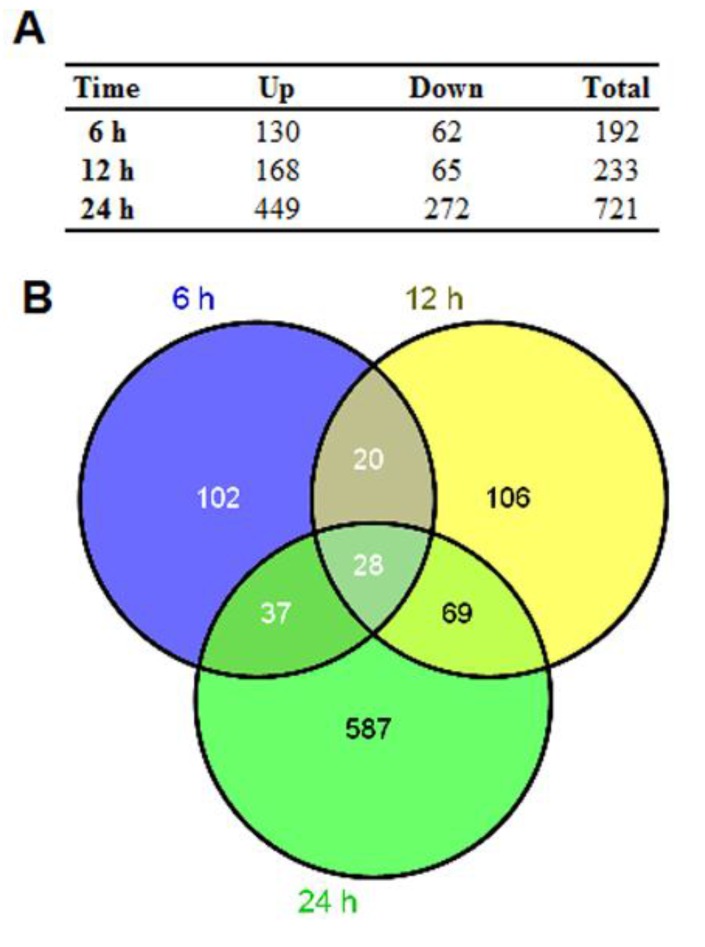
Summary of genes differentially expressed in response to HM/06. (**A**) U251 cells were infected by HM/06 at MOI 1.0. At times 6, 12, and 24 hpi, total RNA was extracted and transcriptional profiles were assessed using 4 × 44K Agilent whole human genome oligo microarray carried by a commercial Agilent array service. Genes with Fold change ≥ 2.0 and *p* < 0.05 in response to HM/06 infection compared with control were indentified to be differentially expressed; (**B**) Over-lapping DE genes at each time post-infection were analyzed by VENNY 2.0.

**Figure 3:**
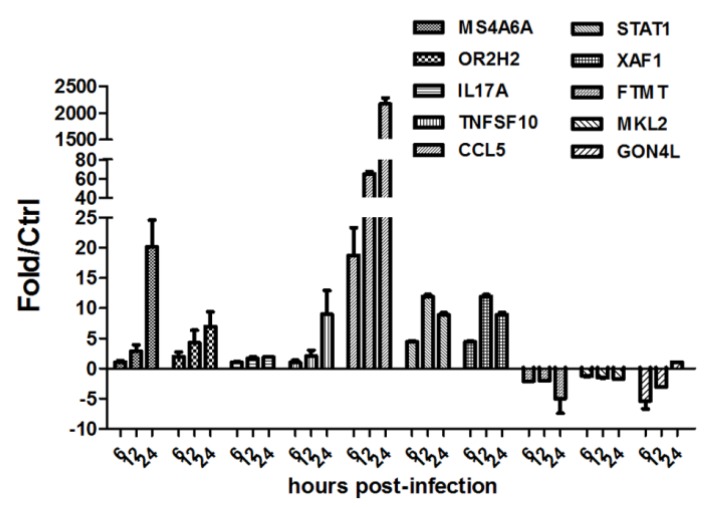
Validation of the microarray data by qRT-PCR. Ten genes with different expressions were selected for RT-PCR confirmation. GAPDH was used for normalization. Data was mean ± SEM of triplicate reactions for each gene. Fold change was calculated using the comparative Ct method. Statistical significance from control infection was analyzed by student’s *t*-test. * *p* < 0.05, ** *p* <0.01, *** *p* < 0.001.

To understand the whole cellular response of U251 to H5N1 infection, Agilent microarray platform was used to compare global gene expression profiles of U251 infected with H5N1, and control. Based on virus replication character (Figure 1A, B, and C), U251 cells were infected with HM/06, at 6, 12, and 24 hpi, RNA was extracted for microarray experiment. Viral infection resulted in significant alterations of mRNA levels at each post-infection time (Figure 2A). Notably, DE genes at 24 hpi dramatically increased up to 721, among which 449 were remarkably upregulated. There were only 28 co-regulated DE genes (Figure 2B) among three post-infection times, with 18 co-upregulated (Supplementary Table S3). Ten genes with different expressions were selected for qRT-PCR confirmation, which showed results consistent with microarray (Figure 3A).

**Figure 4:**
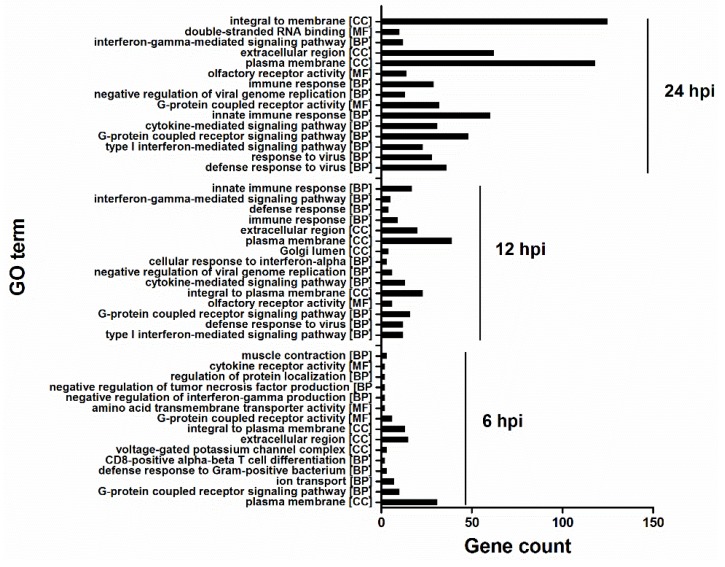
Significantly enriched Gene Ontology (GO) terms according to InnateDB. Gene count refers to DE genes in identified GO terms. Many categories shared the same genes. In addition, only the top 15 were shown based on the number of DE genes in GO terms.

**Figure 5:**
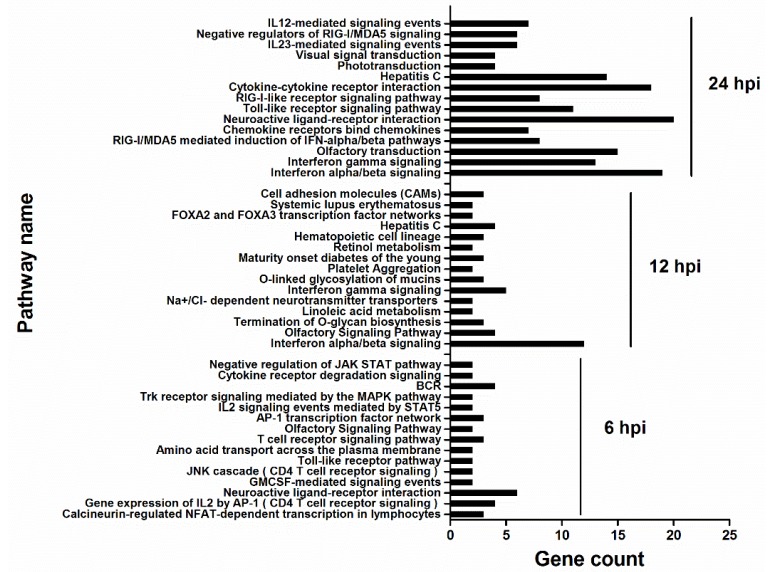
Significantly enriched pathways according to InnateDB. Gene count refers to DE genes in identified pathways. Many categories shared the same genes. In addition, only top 15 were showed based on the number of DE genes in pathways.

To analyze different cellular responses between H5N1 and control infection, Gene Ontology (GO) and pathway over-representation analysis were performed using the tools provided in InnateDB (www.innatedb.ca). The expression data was submitted to InnateDB, and a list of GO terms (Figure 4) and pathways (Figure 5) that were found to be significantly enriched, were generated. There were substantial differences among each infection group. Only three were common to all groups. However, 11 were common to cellular responses at 12 hpi and 24hpi. At 6 hpi, significantly enriched GO terms mainly contained G-protein coupled receptor signaling pathway and ion transport. At 12 and 24 hpi, multiple GO terms involved in immune response, cytokine-mediated signaling, and G-protein coupled receptor signaling pathway were significantly enriched. Notably, though there are many commonly enriched GO terms with 12 hpi, the gene count in enriched GO terms greatly increased at 24 hpi, indicating that at 24hpi, more significant cellular responses were altered with deep infection.

At the pathway level, many pathways were enriched at 6 hpi, including Toll-like receptor pathway (Figure 5). However, like the GO terms enriched at 12 and 24 hpi, the gene count in the enriched pathway significantly increased, particularly at 24 hpi. Interferon alpha/beta signaling was enriched at 12 hpi with 12 upregulated genes, which increased to 19 at 24 hpi. At 24 hpi, in addition to the IFN response pathways, the pathogen pattern-recognition receptor (PRR) pathways (Toll-like receptor and RIG-I-like receptor signaling pathways), and inflammatory response related pathways (chemokine receptors binding chemokines and cytokine-cytokine receptor interaction) were also significantly enriched, suggesting that immune responses were greatly activated to combat viral infection.

### 3.3. HM/06 Infection Triggered a Strong Innate Immune Response

**Figure 6:**
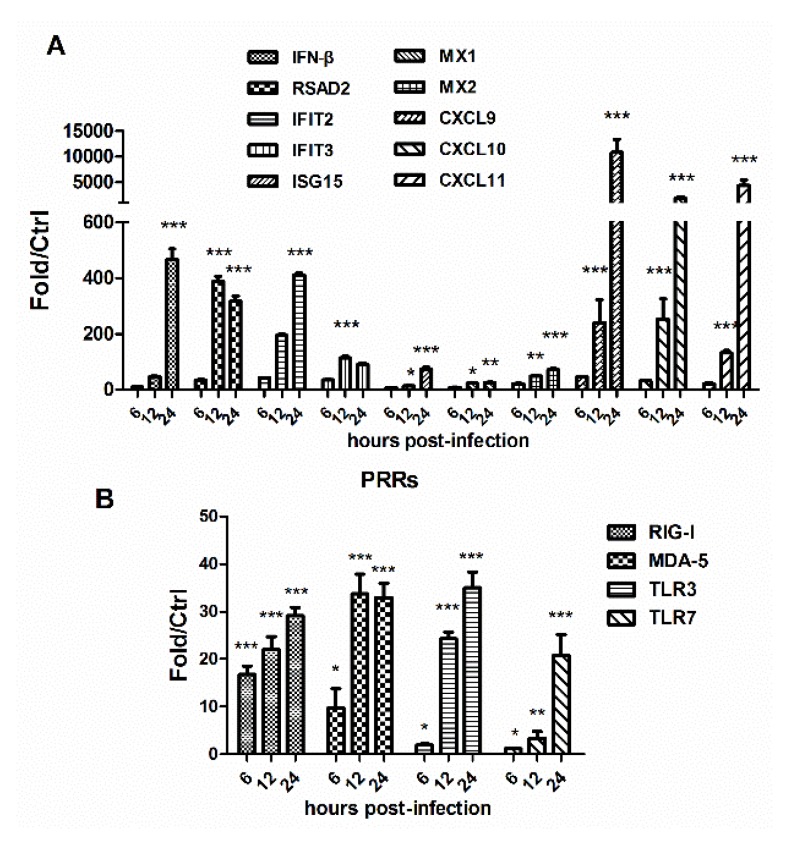
Changes of genes associated with IFN pathway in response to virus infection by qRT-PCR analyses. (**A**) A set of antiviral genes and ISGs were tested; (**B**) PRRs change in response to virus infection. GAPDH was used for normalization. Data was mean ± SEM of triplicate reactions for each gene. Fold change was calculated using the comparative Ct method. Statistical significance of the control infection was analyzed by student’s *t*-test. * *p* < 0.05, ** *p* <0.01, *** *p* < 0.001.

**Figure 7:**
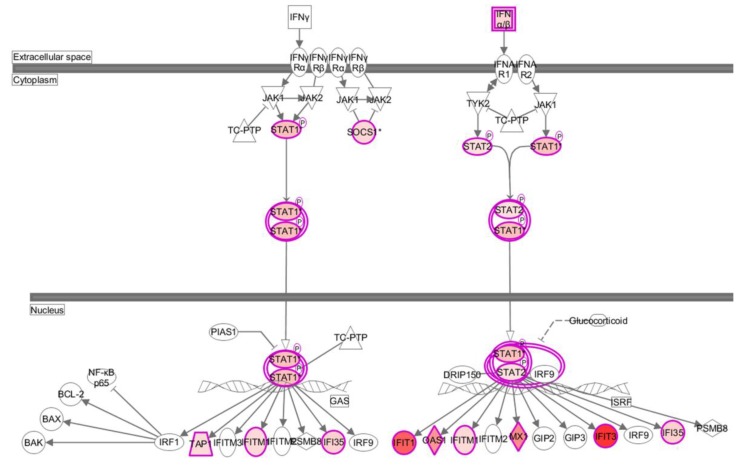
IFN pathway in response to virus infection at 24 hpi. DE genes were submitted to IPA, and IFN pathway in response to virus infection at 24 hpi was constructed. The size and color of nodes reflect the degree of expression difference between control and H5N1 infection. Nodes in red color indicate upregulated genes; Nodes in white color indicate insignificantly changed genes.

At 12 hpi, interferon alpha/beta signaling pathway was significantly enriched (Figure 5). In contrast, at 24 hpi, more pathways involved in innate immune response were significantly enriched, including interferon alpha/beta/gamma signaling and Toll-like/RIG-I-like receptor signaling. Innate immune response is critical in containing viral infection. When activated, many genes, such as interferon stimulated genes (ISGs), well-known PRRs genes associated with IFN production, and other antiviral genes, would afford protection against viral invasion. The data showed that 14 out of the top20 upregulated genes at 24 hpi were antiviral genes (Table 1). In stark contrast, at 6 or 12 hpi, only two were among the top 20 upregulated genes. A mass of genes participating in antiviral response were significantly increased at 24 hpi compared with control (Supplementary Table S2). RT-PCR was used to further determine antiviral gene expression upon viral challenge (Figure 6A). The data revealed that innate immune response was activated as early as 6 hpi, although it drastically strengthened at 24 hpi. Viruses initially activate the innate immune system through PRRs, which recognize viral components and are critical for initiation of antiviral immune responses [19,20]. Several key PRRs were thus tested by using RT-PCR (Figure 6B), which all exhibited significant increase upon viral infection. Using IPA, we constructed the IFN pathway in response to H5N1 at 24 hpi (Figure 7). The size and color of nodes reflect the degree of expression difference between control and H5N1 infection at 24 hpi. Signaling components, such as IFNα/β and STAT1/2, represent the upregulated genes in the pathway, while IFN receptors, such as IFNA1 and IFNA2, generally remain unchanged. Increased mRNA of STAT1/2 would result in the upregulation of phosphorylation status, and gave rise to transcriptional up-regulation of ISGs. SOCS1, an inhibitory feedback mechanism of IFN pathway, was strongly upregulated in response to H5N1 at 24 hpi, which may lead to the limitation of IFN signaling.

**Table 1:** The top 20 genes upregulated by HM/06 infection in U251 cells at each time. Genes in bold indicate ISGs.

6 h			12 h			24 h		
Gene Symbol	*p*-Value	Fold Change	Gene Symbol	*p*-Value	Fold Change	Gene Symbol	*p*-Value	Fold Change
FOSB	9.52E-03	9.37	MS4A6A	9.80E-03	7.08	**RSAD2**	8.20E-04	30.71
LRTM2	2.20E-02	7.91	OR5I1	1.05E-02	6.74	**IFIT2**	5.18E-03	20.90
FOS	7.66E-04	5.64	MPL	1.90E-02	6.63	**IFIT3**	4.96E-04	20.53
MS4A6A	7.86E-03	5.28	OR4K17	3.52E-03	5.52	**CXCL10**	1.47E-02	12.73
ZIM3	3.65E-03	5.27	RSAD2	2.70E-03	5.51	**OASL**	2.04E-03	11.12
MYO1G	2.01E-02	5.04	CD34	2.00E-03	5.28	**MX2**	2.62E-03	10.97
OR4C46	3.37E-02	4.88	MYO1G	3.56E-02	4.39	BATF2	8.10E-03	10.93
TFF3	1.46E-02	4.51	SLC6A20	1.69E-03	4.25	**CXCL11**	1.21E-02	10.14
LHX5	2.22E-02	4.48	ZFP57	4.00E-02	4.23	**IFIT1**	6.01E-04	9.99
BEST3	1.22E-02	4.43	OR8D1	1.57E-02	4.13	**DDX58**	3.99E-04	9.55
HTR3B	1.12E-03	4.20	IFIT3	3.71E-03	4.02	**OAS2**	2.87E-03	9.41
HGFAC	1.55E-02	4.09	TFF3	3.67E-02	3.92	**IFI44L**	3.79E-03	7.73
FAM71B	1.56E-02	4.02	TTLL6	2.30E-02	3.9	**RTP4**	3.04E-03	7.25
AEBP1	3.07E-02	3.73	RBFOX1	2.47E-03	3.87	**MX1**	2.28E-03	7.13
LBP	1.13E-02	3.66	ST6GALNAC1	1.79E-02	3.86	FOSB	3.60E-03	6.89
ZG16	9.93E-03	3.66	KSR1	1.83E-02	3.80	**OAS1**	2.72E-03	6.56
TAS2R46	1.72E-02	3.63	SLC24A2	7.03E-03	3.76	VPREB1	3.78E-02	5.91
MRAP	2.57E-02	3.60	RFX4	1.22E-02	3.75	**BST2**	7.65E-04	5.76
MCHR2	3.16E-02	3.58	OR7A10	1.76E-02	3.54	SAMD9	7.71E-04	5.73
MEP1A	7.00E-03	3.32	CDSN	2.07E-03	3.46	**USP18**	9.88E-03	5.53

### 3.4. HM/06 Infection Induces Extensive Inflammatory Response

**Figure 8:**
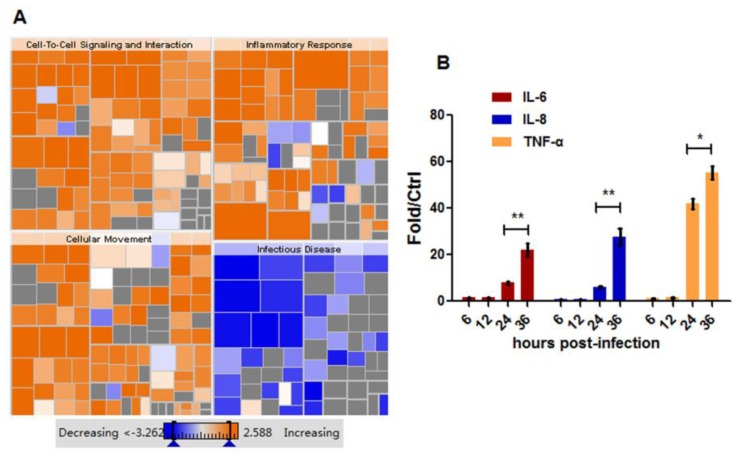
Inflammatory response further strengthened as viral infection deepened. (**A**) Differentially expressed genes at 24 hpi were submitted to IPA and the Downstream Effects Analysis was performed using IPA. Red colors indicate predicted increased biological processes; (**B**) IL-6, IL-8, and TNF-α expression were tested by RT-PCR in response to viral infection. GAPDH was used for normalization. Data was mean ± SEM. Statistical significance was analyzed by student’s *t*-test. * *p* < 0.05, ** *p* < 0.01.

Parallel with activated innate immune response, HM/06 significantly activated the chemokine receptors bind chemokines and cytokine-cytokine receptor interaction pathways at 24 hpi (Figure 5). This observation prompted us to investigate inflammatory response to HM/06 infection. We found that at 6 and 12 hpi, fewer genes related to inflammatory response, significantly increased compared with those at 24 hpi (Table 2). At 24 hpi, increased DE genes included chemokine genes such as CC chemokines CCL5 (3.63-fold) and CCL11 (2.93-fold), as well as CXC chemokines CXCL9-11, which were verified by RT-PCR (Figure 6A). The infection also increased expression of TNF superfamily members 10 (2.39-fold) and 15 (2.34-fold). Finding that inflammatory response to HM/06 was strongest at 24 hpi, we speculated that it would be further strengthened as the virus infected cells more deeply. Therefore, we applied IPA to perform Downstream Effects Analysis, allowing us to easily visualize and explore function hierarchy, and thus envision and predict the effect of gene expression changes on biological processes and trends. As showed in Figure 8A, at 24 hpi, inflammatory response was markedly clustered and predicted to increase. Although in microarray experiment, some important pro-inflammatory cytokines such as TNF-α, IL-6, and IL-8, were not identified to be remarkably changed before 24 hpi, we found that at 36 hpi, the expressions of these cytokines were significantly higher than those at 24 hpi with RT-PCR test (Figure 8B), and exhibited enhanced trend.

**Table 2:** Differentially expressed genes associated with pro-inflammatory response.

hpi	Gene Symbol	Fold Change	*p*-Value
**6**	MPL	2.27	1.35E-02
	IL21	2.63	1.78E-02
	TNFSF8	2.03	3.78E-02
	LEPR	2.54	4.29E-02
	CCR4	2.93	4.76E-02
**12**	IL21	3.23	2.29E-04
	CXCL9	2.21	3.41E-03
	IL17A	2.39	1.58E-02
	MPL	6.63	1.90E-02
**24**	IL17A	3.05	2.75E-03
	INHBC	2.05	2.87E-03
	IL12RB1	2.03	2.94E-03
	CXCL9	5.41	1.07E-02
	CCL5	3.63	1.27E-02
	CXCL10	12.73	1.47E-02
	CCL4	2.11	1.51E-02
	CCR4	2.44	2.27E-02
	IFNB1	2.74	2.35E-02
	LEPR	2.43	2.43E-02
	TNFSF15	2.34	2.62E-02
	IL10RA	2.49	2.80E-02
	IL29	3.17	2.84E-02
	CD274	2.14	3.07E-02
	CTF1	2.20	3.10E-02
	CXCL11	5.32	3.27E-02
	TNFSF10	2.39	3.28E-02
	CCL11	2.93	3.91E-02
	CSF3R	2.08	4.33E-02

**Figure 9:**
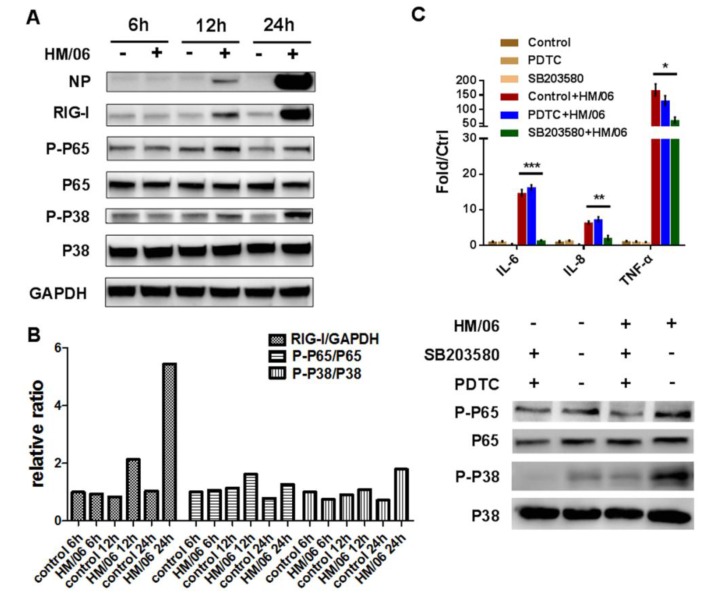
Viral infection increased phosphorylation of p65 and p38 in U251. (**A**) Infected and control cells were lysed in Tris lysis buffer (Cell Signaling) for western blot using corresponding antibodies; (**B**) ImageJ was used for intensity analysis of western blot; (**C**) Cellswere pretreated withNF-κB inhibitor PDTC and p38 kinase inhibitor SB203580 with 100 μM and 5 μM respectively followed by HM/06 infection. At 24 hpi, RNA and protein were collected for qRT-PCR and western blot using corresponding antibodies. For RT-PCR, IL-6, IL-8, and TNF-α were tested with GAPDH as normalization. Data was mean ± SEM. Statistical significance was analyzed by student’s *t* test. * *p* < 0.05, ** *p* < 0.01.

NF-κB and p38 MAPK play key roles in signal transduction and are responsible for a wide range of biological processes such as cytokine synthesis and apoptosis [21,22]. To investigate whether H5N1 infection could activate these signal pathways in U251 cells, we examined the phosphorylation levels of p65 and p38, which are critical for their transcriptional capacity. The phosphorylation of p38 slightly increased at 12 hpi, and significantly increased at 24 hpi (Figure 9A). In contrast, the phosphorylation of p65 highly increased at 12 hpi, and maintained a similar level at 24 hpi compared with control, as calculated with intensity analysis software (Figure 9C).

Considering that RIG-I is of great importance in sensing influenza virus invasion, we measured it upon HM/06 infection (Figure 9A), which showed that RIG-I increased remarkably at 12 hpi, and was much higher at 24 hpi compared with control. These data indicated that NF-κB and p38 MAPK could be important contributors to cytokine activation, and RIG-I plays a significant role in activation of downstream signals in U251 cells. To further investigate the role of NF-κB and p38 MAPK in H5N1-induced downstream cytokines, NF-κB inhibitor PDTC and p38 kinase inhibitor SB203580 were used. The data demonstrated that when cells were pretreated with SB203580, cytokines such as IL-6, IL-8, and TNF-α significantly decreased following virus infection. However, PDTC treatment had no significant influence (Figure 9C). It indicated that the p38 MAPK pathway could play more important role in pro-inflammatory response to H5N1 infection in astrocytes.

### 3.5. H5N1 Modified Internal Ca^2+^and EGTA Treatment Decreased Virus-Induced Apoptosis

**Figure 10:**
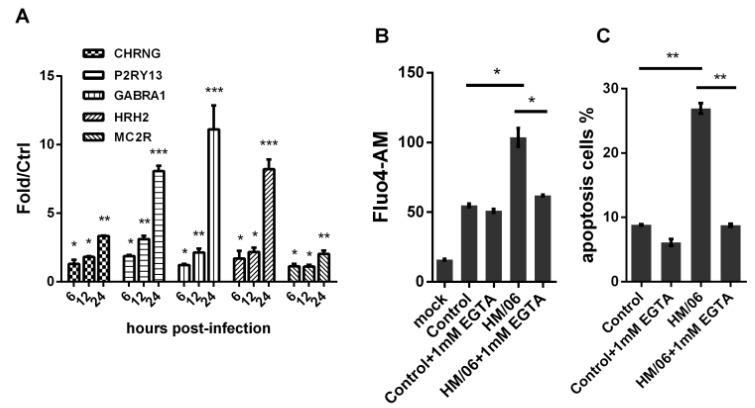
H5N1 infection elevated internal Ca^2+^ and EGTA treatment decreased virus-induced apoptosis. (**A**) H5N1 infection upregulated several genes functioned as receptor for neurotransmitters via qRT-PCR test; (**B**) H5N1-infected cells were treated with control or 1 mM EGTA for 24 h. 5 μM Fluo4-AM in HBSS was added for 30 min, and then Fluo4-AM fluorescence was tested by BD FACSCalibur system; (**C**) Apoptosis of control or EGTA treated infected-cells was analyzed using FITC-annexin V Apoptosis Detection Kit and BDFACSCalibur system. Data was mean ± SEM. Statistical significance was analyzed by student’s *t*-test. *p < 0.05, **p < 0.01, ***p < 0.001.

Astrocytes exert irreplaceable function in regulation of CNS microenvironment and neuronal migration [13]. In microarray test, H5N1 infection induced upregulation of genes associated with neuroactive ligand-receptor interaction pathway at 6 hpi and 24 hpi, with most DE genes at 24 hpi (Table 3). For instance, CHRNG (2.36-fold), P2RY13 (4.5-fold), GABRA1 (2.33-fold), HRH2 (2.12-fold), and MC2R (2.46-fold), were significantly upregulated at 24 hpi, which were confirmed by RT-PCR test (Figure 10A). These genes were reported to be functioned as receptors for neurotransmitters, and their activation could result in oscillation in internal Ca^2+^ [23]. Thus, we investigated Ca^2+^dynamics in U251 cells infected with H5N1. As shown in Figure 10B, infected cells showed marked elevation of Ca^2+^ at 24 hpi compared with control infected cells. Ca^2+^ is a key regulator of cell survival, and breakdown of its homeostasis will trigger programmed cell death involving apoptosis [24]. Disruption of Ca^2+^was reported to play an important role in apoptosis during several types of viral infections, including influenza virus in avian cells [25,26,27]. In this study, EGTA, a chelator of extracellular Ca^2+^, was used to analyze the effect of Ca^2+^ influx on apoptosis of U251 cells infected with H5N1. Reduced level of Ca^2+^ in infected U251 cells were found in presence of EDTA at 24 hpi. In addition, H5N1-induced apoptosis was attenuated in EGTA-treated U251 cells (Figure 10C). It suggested that disruption of Ca^2+^ by H5N1 infection would play a key role in apoptosis in U251 cells.

**Table 3:** Differentially expressed genes in neuroactive ligand-receptor interaction pathway in response to HM/06 infection.

hpi	Gene Symbol	*p*-Value	Fold Change
**6**	VIP	5.28E-03	2.59
	CHRNB1	1.67E-02	2.22
	MCHR2	3.16E-02	3.58
	HCRTR1	3.87E-02	0.49
	MC2R	2.87E-02	2.11
	LEPR	4.29E-02	2.54
**12**	HTR1A	1.03E-03	2.95
	NTSR1	6.50E-03	3.22
**24**	HTR1A	1.86E-03	2.26
	GHRHR	2.60E-03	2.13
	CHRNG	2.78E-03	2.36
	GRM7	4.34E-03	2.01
	SSTR2	5.44E-03	2.48
	CGA	1.07E-02	2.17
	P2RY13	1.09E-02	4.50
	OPRM1	1.11E-02	2.17
	GABRA1	1.25E-02	2.33
	MC3R	1.38E-02	2.96
	CNR2	1.76E-02	2.65
	LEPR	2.43E-02	2.43
	CCKAR	2.77E-02	2.56
	HRH2	3.07E-02	2.12
	MCHR2	3.34E-02	3.04
	MC2R	4.00E-02	2.46
	HTR1B	4.30E-02	2.62
	NPB	4.57E-02	2.28
	MLNR	4.77E-02	2.43
	TAAR8	4.98E-02	2.00

## 4. Discussion

In this study, a functional genomics approach was used to investigate comprehensive host responses to H5N1 infection in U251 cells. We selected HM/06, an highly pathogenic H5N1 virus, because it was isolated in brains of domestic ducks exhibiting neurovirulence [17] and can infect mouse brain. It could efficiently grow in U251 cells and induce significant cytopathy (Figure 1). In the microarray analysis, groups of signal pathways were identified to be activated, among which, innate immune response was desirable. Innate immune response plays an important role in eliminating viral infection. Upon recognizing viral RNA by PRRs, host will initiate IFN pathway, leading to transcriptional induction of ISGs, which is a formidable barrier against viral multiplication in infected host. Many genes related to IFN signal were in the top20 significantly upregulated genes at 24 hpi compared with control (Table 1). Notably, at 6 hpi, fewer genes were remarkably altered upon infection. This finding was in marked contrast with a previous study performed in human alveolar macrophages infected by PR/8, which showed that at 4 hpi, many genes related to IFN were significantly increased [28]. This indicates that there are big differences between alveolar macrophages and astrocytes in their properties of activating innate immune response to influenza virus, including intensity and time. Interestingly, genes associated with neuroactive ligand-receptor interaction pathway, which included VIP, MCHR2, MC2R, and CHRNB1, is most significantly regulated at 6 hpi, suggesting that at early stage of virus replication, neuron signaling transduction might be modulated by virus.

It is well known that RIG-I like receptors (RLRs) and TLRs are the main PRRs responsible for recognizing molecular patterns of microorganisms and IFN production against RNA viruses, including influenza [19,20,29,30,31]. In the current study, H5N1 infection increased mRNA of RIG-I, MDA-5, TLR3, and TLR7 (Figure 6B). Interestingly, RIG-I and MDA-5 increased to a higher change than TLR3 and TLR7 at 6 hpi, suggesting RLRs might play more vital roles in sensing RNA at early stage of influenza infection than TLRs. IFNs are pivotal in antiviral response. Along with increased PRRs gene expression, IFN-β was identified to be upregulated, as well as IL29 in microarray test. However, IFN-γ, a member of type III IFN, was not significantly changed. Among the upregulated anti-viral genes, RSAD2 is an evolutionary conserved protein, which can be highly induced by IFNs [32]. It was reported that it can inhibit influenza virus release in an *in vitro* study [33]. Notwithstanding this, an *in vivo* study demonstrated that RSAD2 did not confer protection against H1N1 [34]. However, it is noteworthy that RSAD2 could protect against SINV and WNV, which cause acute inflammation in the CNS [35,36], suggesting that in CNS, a relatively sterile environment, RSAD2 may exert critical role in combating viral infection. In U251 cells, H5N1 infection significantly increased the expression of RSAD2. H5N1, unlike H1N1, can enter CNS and lead to inflammation in most cases. Therefore, it is greatly appealing to investigate whether RSAD2 could play a role in preventing escalating H5N1infection, particularly in CNS. IFIT family members (IFIT1,2,3, and 5), were among the top20 genes upregulated by H5N1 infection at 24 hpi in U251 (Table 1). IFITs are highly induced ISGs in response to IFN or viral infection [37]. Deficiency of IFIT expression, particularly IFIT1, conferred advantage to viral growth [38], albeit forced expression could not or only modestly help to inhibit virus replication [38,39,40,41]. In addition, OAS1, OAS2, MX1, MX2, IFI44L, GBP1, IFITM1, and BST2, also increased to varying degrees, suggesting an activated host immune state at 24 hpi. Remarkably, ISG15, ISG56, GBP4, and USP18, which had been reported to be negative regulators of IFN signaling [42,43,44,45], were identified to be upregulated at 24 hpi. These findings indicated that the upregulated inhibitory genes were equally important for protecting host, by restricting over-activated host innate immune response, other than directly limiting the virus.

Previous studies suggest that influenza infection induces more immunological activity than necessary to eliminate the viruses, designating it “cytokine storm,” characterized by an excessive or uncontrolled release of pro-inflammatory cytokines [46]. *In vitro* and *in vivo* evidence demonstrate that the unusual severity of H5N1 is associated with intense inflammatory responses [15,47,48]. In the microarray analysis, we observed that many pathways associated with inflammatory response were over-represented, particularly at 24 hpi, such as chemokine receptors bind chemokines and cytokine-cytokine receptor interaction. Many chemokines were significantly upregulated at 24 hpi, among which, CXCL9-11 was of great interest. These chemokines bind to CXCR3, signaling mediated by which has been revealed in the pathogenesis of influenza [49]. Although these are IFN-induced genes, and likely contributing to antiviral response, overproduction would be detrimental to the host. In fact, blocking CXCR3 by AMG487 can alleviate severe symptoms and delay mortality induced by H5N1 [50]. This suggests that CXCL9-11 would play a vital role in the neuropathogenesis of H5N1. Additionally, CCL4, CCL5, and CCL11 remarkably increased. Surprisingly, some cytokines reported to be hyper-induced by H5N1in primary human macrophages and respiratory epithelial cells, which may contribute to H5N1 pathogenesis, were insignificantly changed in microarray analysis. This finding may be ascribed to the differences in the nature of pro-inflammatory response in macrophages, epithelia, and astrocytes. However, when we performed the Downstream Effects Analysis using IPA, we observed that inflammatory response further strengthened as viral infection deepened, which encouraged us to investigate cytokine expressions at relatively late post-infection. As expected, at 36 hpi, IL-6, IL-8, and TNF-α increased significantly compared with those at 24 hpi. It demonstrated that HM/06 can substantially induce inflammatory response that will cause immunologic injury to U251 cells.

To investigate which pathway is involved in induction of pro-inflammatory cytokines, phosphorylation levels of p65 and p38 were examined (Figure 9A), which showed that both can be significantly activated by H5N1. The IL-6, IL-8, and TNF-α were significantly reduced when cells were pretreated with p38 kinase SB203580 rather than NF-κB inhibitor PDTC (Figure 9C), suggesting that p38 pathway may play more important role in pro-inflammatory response to H5N1. It was reported that induction of pro-inflammatory cytokines by H5N1 was selectively regulated by IRF3 and p38 MAPK in macrophages [51]. However, details of induced pro-inflammatory response in astrocytes still remain unknown. Future research should focus on this, to reinforce our understanding of pro-inflammatory response mechanisms induced by H5N1 in CNS.

Astrocytes play critical roles in supporting neuronal function. They operate hand in hand with neurons to regulate integration in the CNS. Significantly, astrocytes express a plethora of receptors for many neurotransmitters [52,53], and their activation result in oscillations in internal Ca^2+^, which then induce accumulation of arachidonic acid and release of chemical transmitters such as glutamate, D-serine, and ATP [54]. These receptors include GABA, acetylcholine, histamine, and adenosine, which have all been shown to induce Ca^2+^ elevation. It was also discovered that chemical transmitters can evoke Ca^2+^ elevations in astrocytes 23]. Thus, astrocytes are appreciated to ‘listen’ and ‘talk’ to synapses, modulate synapses, and mediate synaptic cross-talk [55]. In this study, many genes associated with neuroactive ligand-receptor interaction pathway significantly increased at 24 hpi, including those functioning as receptors for neurotransmitters, such as MC2R, CHRNG, P2RY13, GABRA1, and HRH2 (Table 3 and Figure 10A). Therefore, we tested internal Ca^2+^ in H5N1-infected U251 cells, which showed that H5N1 infection can significantly elevate internal Ca^2+^ (Figure 10B). When internal Ca^2+^ was decreased by EGTA, virus-induced apoptosis significantly reduced (Figure 10C). This indicated that H5N1 can modulate internal Ca^2+^ dynamic in astrocytes, which may play important role in apoptosis. Interestingly, although significant apoptosis was found in H5N1-infected astrocytes, we did not identify important changed genes related to apoptosis. It might be due to that apoptosis is very complex process involving protein translocation, signaling activation, protein cleaving, and other factors such as internal Ca^2+^ dynamic except direct transcription and translation, or that genes related to apoptosis were not upregulated to the threshold value of array detecting.

However, there is considerable signaling exchange between cell types within CNS, making it more complicated to elucidate key factors associated with CNS disorder in a single cell line alone. Further studies should be performed using co-culture cells such as astrocyte-neuron and microglia-neuron, or using the brain as a model to get a more clear view on mechanism of H5N1-induced CNS disorder and neuropathology.

Another topic of widespread concern is how influenza virus enters the CNS. Although reported data pointed out that H5N1 reaches the CNS by nerve routes [56,57], alteration of BBB integrity by virus may also be a route for invasion. Considering that astrocytes are one of the cell types constituting BBB, once they are damaged by the virus, BBB could be disrupted, and become susceptible to peripheral virus entry. From this perspective, astrocytes exert a significant role in protecting CNS. More attention should be focused on the interaction between influenza virus and BBB.

Overall, our study looked into global responses of U251 cells to H5N1 infection by microarray analysis, and provided detailed information, which can contribute to the understanding of H5N1pathogenesis in astrocytes and mechanism of H5N1-induced CNS disorders.

## 5. Conclusions

In short, we explored global responses of human astrocytes to H5N1 infection through transcriptional analysis for the first time, which suggested that over-activated IFN signaling and excessive pro-inflammatory responses could contribute to the immunologic injury of U251 by H5N1 infection, and that the p38 MAPK pathway may play important role in pro-inflammatory response to H5N1 infection. Moreover, H5N1 infection could modify genes expression associated with neuroactive ligand-receptor interaction and internal Ca^2+^ dynamic, which could be related to the neural signaling disorder and apoptosis caused by H5N1 infection.

## Supplementary Files

Supplementary File 1
