# Supplementary material for: Insights into Human Astrocyte Response to H5N1 Infection by Microarray Analysis
Source: Viruses. 2015 May 22;7(5):2618–40. doi: 10.3390/v7052618 (PMC4452922; doi:10.3390/v7052618)
Supplement: Supplementary File 1 [file viruses-07-02618-s001.pdf]

Supplementary Materials

Table S1. Primers used in this study.

Genes	Forward Sequences	Reverse Sequences
RIG-I	GTGCAAAGCCTTGGCATGT	TGGCTTGGGATGTGGTCTACTC
MDA5	GTTGAAAAGGCTGGCTGAAAAC	TCGATAACTCCTGAACCACTG
TLR3	CTCAGAAGATTACCAGCCGCC	CCATTATGAGACAGATCTAATG
TLR7	CACATACCAGACATCTCCCCA	CCCAGTGGAATAGGTACACAGTT
CXCL9	CCAGTAGTGAGAAAGGGTCGC	AGGGCTTGGGGCAAATTGTT
CXCL10	TGGCATTCAAGGAGTACCTCTC	CTTGATGGCCTTCGATTCTG
CXCL11	GACGCTGTCTTTGCATAGGC	GGATTTAGGCATCGTTGTCCTTT
IFIT2	GACACGGTTAAAGTGTGGAGG	TCCAGACGGTAGCTTGCTATT
IFIT3	AGAAAAGGTGACCTAGACAAAGC	CCTTGTAGCAGCACCCAATCT
MX1	GGTGGTCCCCAGTAATGTGG	CGTCAAGATTCCGATGGTCCT
ISG15	CGGGAACAAGTCCACGAA	CAACACTGGCTCTGGATGG
MX2	CAGAGGCAGCGGAATCGTAA	TGAAGCTCTAGCTCGGTGTTC
RSAD2	TTGGACATTCTCGCTATCTCCT	AGTGCTTTGATCTGTTCCGTC
IL-6	AGGAGACTTGCCTGGTGAAA	CAGGGGTGGTTATTGCATCT
IL-8	TTGGCAGCCTTCCTGATTTC	TCTTTAGCACTCCTTGGCAAAAC
CCL5	CCTCATTGCTACTGCCCTCT	GGTGTGGTGTCCGAGGAATA
GAPDH	GCAAAGGCTGTGGGCAAGG	GGAGGAGTGGGTGTCGCTG
TNF-α	ACGGCATGGATCTCAAAGACAACC	TGAGATAGCAAATCGGCTGACGGT
IFN-β	GCTTGGATTCCTACAAAGAAGCA	ATAGATGGTCAATGCGGCGTC
GABRA1	AGCCGTCATTACAAGATGAACTT	TGGTCTCAGGCGATTGTCATAA
P2RY13	ATCGTGCTGTTAGGGCTCATA	CAAGATCGTATTTGGCAGGGAG
MC2R	GACTGTCCTCGTGTGGTTTTG	GGCTGCCCAGCATATCAGAT
CHRNG	CTCCTCTGTCGCCATCCTCA	GGAAGGTCAGGTACTTGCTGAT
HRH2	CAGCAAGGGCAATCATACCAC	GATCAGTAGCGGGAGGTAGAA
MS4A6A	TGTGGCATGATGGTATTGAGC	AGGGTCCTATGAATGGGTAAGC
OR2H2	CTTGTCTCTTACGGAGCCATTAC	TCTCTTGGGCATAGGGATTTTTG
XAF1	GCTCCACGAGTCCTACTGTG	GTTCACTGCGACAGACATCTC
STAT1	CGGCTGAATTTCGGCACCT	CAGTAACGATGAGAGGACCCT
FTMT	TGGAGTGTGCTCTACTCTTGG	ACGTGGTCACCTAGTTCTTTGA
GON4L	CTCAGGGAATCACCCTACCTT	CTCAGGGAATCACCCTACCTT
IL17A	TCCCACGAAATCCAGGATGC	GGATGTTCAGGTTGACCATCAC
MKL2	TTATAGGCGTTGGGAAGGAGG	CCGGAGACAAAGCGTCACT
TNFSF10	TGCGTGCTGATCGTGATCTTC	GCTCGTTGGTAAAGTACACGTA

Table S2. Upregulated antiviral genes at 24 hpi.

Gene Symbol	Fold Change	*p*-Value
RSAD2	30.71	8.20E-04
IFIT2	20.90	5.18E-03
IFIT3	20.53	4.96E-04
OASL	11.12	2.04E-03
MX2	10.97	2.62E-03
IFIT1	9.99	6.01E-04
DDX58	9.55	3.99E-02
OAS2	9.41	2.87E-03
IFI44L	7.73	3.79E-03
MX1	7.13	2.28E-03
OAS1	6.56	2.72E-03
IFIH1	5.12	1.57E-03
ISG15	4.46	1.82E-03
OAS3	4.05	2.02E-03
STAT1	3.97	3.06E-03
XAF1	3.82	1.09E-02
IFI6	3.79	4.38E-03
GBP1	3.60	2.54E-02
IFIT5	3.57	4.24E-03
DHX58	3.19	2.90E-03
IL29	3.17	2.84E-02
IFI27	3.13	3.65E-03
IFI35	2.92	4.53E-02
GBP4	2.90	1.10E-02
IFNB1	2.74	2.35E-02
IRF7	2.65	1.24E-02
ZBP1	2.60	5.87E-03
TAP1	2.41	4.00E-02
IFITM1	2.38	1.41E-02
TLR3	2.37	3.03E-02
IFI16	2.18	6.38E-03
MYD88	2.13	2.60E-03
ADAR	2.12	3.37E-03
STAT2	2.01	2.38E-03

Table S3. Co-upregulated genes among each time post-infection.

Gene Symbol	6 h		12 h		24 h	
	p-Value	Fold Change	*p*-Value	Fold Change	*p*-Value	Fold Change
MS4A6A	7.86E-03	5.28	9.80E-03	7.08	3.22E-03	2.57
TFF3	1.46E-02	4.51	3.67E-02	3.92	1.26E-02	3.05
ZG16	9.93E-03	3.66	7.51E-03	2.69	4.18E-02	2.77
MEP1A	7.00E-03	3.32	2.33E-02	2.23	1.30E-02	2.39
MAB21L3	1.97E-02	3.15	9.85E-03	3.46	2.82E-02	2.66
RFX4	2.07E-02	3.14	1.22E-02	3.75	3.78E-02	4.94
DEFB123	5.74E-03	2.86	1.73E-03	3.42	3.62E-02	4.34
POPDC2	4.28E-02	2.77	1.77E-03	3.08	1.99E-02	3.79
CDSN	3.27E-02	2.61	2.07E-03	3.46	6.29E-03	2.39
OR2H2	1.65E-02	2.27	1.27E-02	2.45	1.42E-02	4.92
ZFP57	1.81E-02	2.22	4.00E-02	4.23	2.11E-03	2.65
MUC5AC	1.85E-02	2.19	2.49E-02	2.10	1.58E-02	2.13
APOA5	5.41E-03	2.18	1.03E-02	2.41	9.82E-03	2.09
CD55	7.95E-03	2.14	9.78E-03	2.21	3.23E-02	2.23
RNF39	3.36E-02	2.11	3.51E-03	3.20	2.77E-02	2.66
GAL3ST2	2.54E-02	2.10	1.38E-02	3.28	1.46E-02	4.75
CSHL1	2.64E-03	2.07	5.84E-04	2.03	4.61E-02	2.51
MUC12	3.58E-02	2.04	2.54E-02	2.05	2.83E-02	3.71
